# Case report: Compound heterozygous *NUP85* variants cause autosomal recessive primary microcephaly

**DOI:** 10.3389/fneur.2023.1124886

**Published:** 2023-02-09

**Authors:** Ethiraj Ravindran, Gaetan Lesca, Louis Januel, Linus Goldgruber, Achim Dickmanns, Henri Margot, Angela M. Kaindl

**Affiliations:** ^1^Institute of Cell Biology and Neurobiology, Charité – Universitätsmedizin Berlin, Berlin, Germany; ^2^Department of Pediatric Neurology, Charité – Universitätsmedizin Berlin, Berlin, Germany; ^3^Center for Chronically Sick Children (Sozialpädiatrisches Zentrum, SPZ), Charité – Universitätsmedizin Berlin, Berlin, Germany; ^4^Department of Genetics, Hospices Civils de Lyon, Groupe Hospitalier Est, Bron, France; ^5^Institut NeuroMyoGene PNMG, CNRS UMR5310, INSERM U1217, Université Claude Bernard Lyon 1, Lyon, France; ^6^Department of Biomedical Engineering, Veterinärmedizinische Universität (Vetmeduni), Vienna, Austria; ^7^Department of Molecular Structural Biology, Institute for Microbiology and Genetics (GZMB), Georg-August-University Göttingen, Göttingen, Germany; ^8^Department of Medical Genetics, University of Bordeaux, MRGM INSERM U1211, CHU de Bordeaux, Bordeaux, France

**Keywords:** NUP85, microcephaly, brain development, speech disorder, MCPH-SCKS

## Abstract

Nucleoporin (NUP) 85 is a member of the Y-complex of nuclear pore complex (NPC) that is key for nucleocytoplasmic transport function, regulation of mitosis, transcription, and chromatin organization. Mutations in various nucleoporin genes have been linked to several human diseases. Among them, NUP85 was linked to childhood-onset steroid-resistant nephrotic syndrome (SRNS) in four affected individuals with intellectual disability but no microcephaly. Recently, we broaden the phenotype spectrum of NUP85-associated disease by reporting *NUP85* variants in two unrelated individuals with primary autosomal recessive microcephaly (MCPH) and Seckel syndrome (SCKS) spectrum disorders (MCPH-SCKS) without SRNS. In this study, we report compound heterozygous *NUP85* variants in an index patient with only MCPH phenotype, but neither Seckel syndrome nor SRNS was reported. We showed that the identified missense variants cause reduced cell viability of patient-derived fibroblasts. Structural simulation analysis of double variants is predicted to alter the structure of NUP85 and its interactions with neighboring NUPs. Our study thereby further expands the phenotypic spectrum of NUP85-associated human disorder and emphasizes the crucial role of NUP85 in the brain development and function.

## Introduction

Nucleoporin (NUP) 85 is a member of the Y-complex of nuclear pore complex (NPC) that is key for nucleocytoplasmic transport function (1). Along with NUP85, other members of the Y-complex (NUP160, NUP133, NUP107, NUP96, NUP43, NUP37, SEH1, and SEC13) are also known to regulate mitosis, transcription, and chromatin organization (2, 3). Downregulation of NUP107-160 subcomplex members resulted in defective cytokinesis, compromised microtubule structures, altered cytoskeletal dynamics, and impaired chromosome segregation and differentiation (4–6). Variants in several genes encoding NUP components have been linked to the spectrum of human disease (Supplementary Table S1, Supplementary material) (7). NUP85 was initially linked to childhood-onset steroid-resistant nephrotic syndrome in four affected individuals (SRNS) (MIM^*^618176) with intellectual disability (ID) but neither microcephaly nor brain malformation (4). Recently, we reported biallelic *NUP85* variants in two unrelated individuals with primary autosomal recessive microcephaly (MCPH) and Seckel syndrome (SCKS) spectrum disorders (MCPH-SCKS) without SRNS and thereby broaden the phenotype spectrum of NUP85-associated diseases (8). Here, we report compound heterozygous *NUP85* variants in a child with MCPH, but without the short stature seen in Seckel syndrome.

## Materials and methods

### Patients

Written informed consent was obtained from the parents of the index patient for participation in the study, molecular genetic analysis, and publication. The human study was approved by the local ethics committees of the DEFIDIAG project (the pilot project of the Plan France Genomique 2025).

### Genetic analysis

The whole genome sequencing trio analysis was carried out within the framework of the DEFIDIAG project (the pilot project of the Plan France Genomique 2025) (Supplementary material).

### Fibroblast culture

Fibroblast culture was established using the explant technique from the index patient and the unrelated controls were cultured in high-glucose Dulbecco's modified Eagle's medium (DMEM GlutaMAX supplement pyruvate, Gibco, Paisley, Scotland) with 10% fetal bovine serum (FBS, Gibco, Paisley, Scotland), and 1% penicillin/streptomycin (P/S, Gibco, Grand Island, the USA) at 37°C.

### Western blot

Protein extraction and Western blots were performed in triplicates with the established methods reported previously (9). The antibodies used in this study were anti-NUP85 (Proteintech, rabbit) and anti-actin (Millipore, mouse).

### Cell viability assay

Fibroblasts of the patient and controls were seeded at a density of 10^3^ cells/well in 96-well plates. Cell viability (fluorimetric CellTiter-Blue Cell Viability Assay^®^, Promega, Madison, the USA) was performed according to the manufacturer's instructions as described previously (9), readings were measured using a SpectraMax iD3 plate reader (Molecular devices, San Jose, the USA), and data were analyzed using GraphPad Prism 6 Software (version 6.07) (GraphPad Software Inc., La Jolla, CA, the USA).

### Structural analysis of NUP85

The PDB has been searched for human Nup85 wild-type structure (sequence Q9BW27 from UniProt). This search resulted in the identification of the currently best resolved (12 A) electron microscopy structure of the human nuclear pore complex (PDB id 7R5K) with Nup85 being annotated as entity number 18. The atomic model of Nup85 has been extracted from that coordinates file and subjected to homology modeling of its double mutant (Leu152Ile/Leu163Ile) structure using the comparative modeling approach as implemented in the ROSETTA package (10). In order to more accurately model the bulkier Ile residues located in a crowded environment, a short fragment, namely, region 151–164, accommodating both mutated residues (Leu152Ile and Leu163Ile) has been deleted and “*de novo*” remodeled using Rosetta's loop building algorithms. Fragment libraries required for protein structure prediction have been obtained from the Robetta server (http://robetta.bakerlab.org). Over 1,000 homology models have been generated, which have been assessed based on the Rosetta energy score. The model with the lowest (best) score has been selected as the homology model for further analysis. The contacts of the residues 152 and 163 either in wild type or double mutant were analyzed using Arpeggio under standard settings (11). Structural interpretation of either Nup85 alone or in a complex within the NPC using PDBid: 7r5k, 7tbl, and 7peq were performed (12–14). The figures were generated using PyMOL (Schrödinger LLC).

## Results

**Figure 1:**
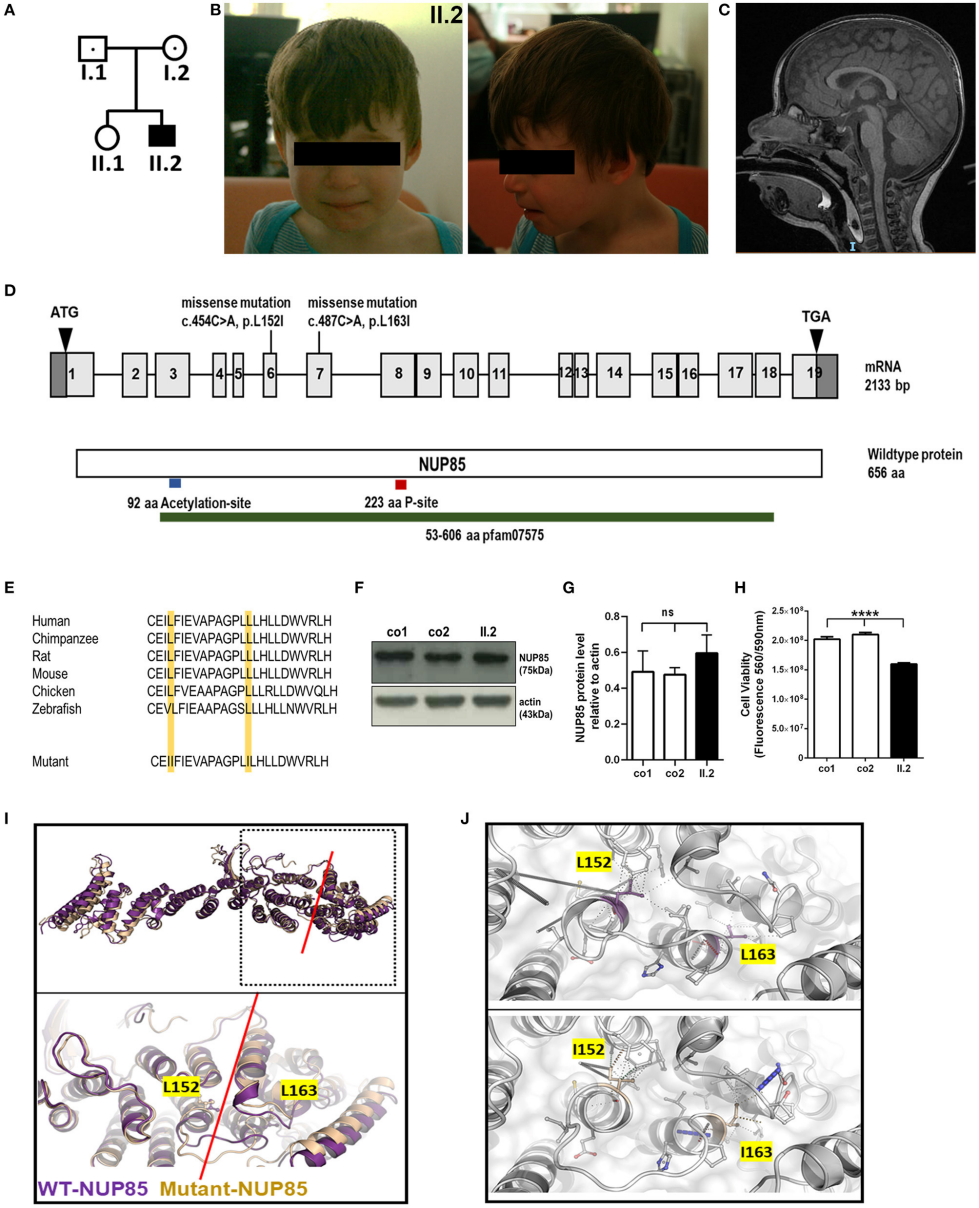
Phenotype of index patient with compound heterozygous *NUP85* mutation. **(A)** Pedigree. **(B)** Pictures of affected individual (II.2). **(C)** MRI images of the head (sagittal) T2 images of II.2 shows reduced brain volume. **(D)** Representation of identified compound heterozygous variants by whole genome sequencing in the *NUP85* cDNA c.454C > A in exon 6 (maternally inherited) and c.487C > A in exon 7 (paternally inherited) and NUP85 wild-type protein (p.L152I, p.L163I). **(E)** Mutations lie in the highly conserved region of the NUP85 protein across species. **(F)** Unchanged levels of total NUP85 protein in II.2-derived fibroblasts compared to controls [NUP85 (75kDa), actin (43kDa) (loading control)] (*n* = 3, one-way ANOVA, Tukey's multiple comparison test, *p* = 0.2973). **(H)** Cell viability in II.2-patient derived fibroblasts is significantly reduced compared to controls (n=8, one-way ANOVA, Tukey's multiple comparison test, ****p < 0.0001). **(I)** Structural overlay of human NUP85 based on PDBid 7R5K (purple) and the L152I/L163I double mutant (wheat). Top panel: Overlay of the overall structures; bottom panel: magnification of the hinge region (indicated by red line), the structures have been overlaid using the central region of the molecules. The mutations are located in this hinge region and are indicated in ball and stick mode. **(J)** Significant change of the interaction pattern due to mutations. Magnification of the interaction patterns as evaluated using the program Arpeggio under standard settings. Top panel wild-type, bottom panel-double mutant. The mutated residues are indicted in the colors as in **(I)**.

The index patient was recruited as part of the DEFIDIAG Study Group. The index patient (II.2) was a 3.6-year-old boy born at term as a second child to non-consanguineous parents without complications (Figures 1A, B). His body weight and length were normal at birth [2,740 g (3rd centile) and 49 cm (10th centile)]. Primary microcephaly was already severe at birth with the occipitofrontal head circumference (OFC) of 31 cm [−3 standard deviations (SD), <3rd centile] (Supplementary Figure S1, Supplementary material) (Table 1). At the age of 3 years, the OFC was 46 cm (<-3 SD), while the weight (11 kg, −2 SD) and height (86 cm, −1 SD) were normal (Supplementary Figure S1). The boy displayed facial dysmorphism (almond-shaped eyes, simplified ears, short philtrum) and *Pes adductus* (Figure 1B). He had a global developmental delay with a pronounced speech disorder. He could not speak until the age of 2 years. He was able to say disyllabic words starting at 2 years and 50 words at 3.6 years of age without the proper frame of the sentences. Psychomotor evaluation at 22 months showed −4 SD for posturomotor and locomotor scores and a −6 SD for grip and visuomotor coordination score. He communicated preferably through eye contact and pointing at objects. Motor milestones were normal with free ambulation at 17 months of age. Fine motor skills were delayed with pincer grip at 14 months. He displayed hyperactivity, repetitive behavior, a frustration intolerance, and hetero-aggressive behavior. He is really selective about food. An ophthalmological examination showed esophoria and astigmatism at the last follow-up. Cranial MRI at 1.4 years of age revealed reduced global brain volume and delayed myelination (Figure 1C). Electroencephalograph data were normal. He has a sleeping disorder with a short night span and multiple awakenings, despite the intake of melatonin. He displayed abnormal movement during sleep. He started mainstream school part time with a specialized classroom assistant and made constant progress.

**Table 1:** Clinical features of individuals with *NUP85* mutations.

**Characteristics and symptoms**	**Index patient**	**Patient 1 (P1)^a^**	**Patient 2 (P2)^a^**	**Patient 3 (A5195-22^a^)^b^**	**Patient 4 (A3259-21)^b^**	**Patient 5 (NCR3227)^b^**	**Patient 6 (NCR3310)^b^**
NUP85 variant (NM_024844.5)	c.454C > A, c.487C > A	c.932G > A	c.1109A > G, c.1589T > C	c.1430C > T	c.1933C > T	c.405 + 1G > A	c.1741G > C
Parents consanguinity	–	+	–	+	–	–	–
Sex	Male	Female	Female	Female	Male	Female	Male
Age at last assessment	3.6 years	9 years	27 GW	8 years	11 years	7 years	4 years
Age at onset	birth	birth	prenatal	8 years	11 years	7 years	4 years
Primary microcephaly	+	+	+	NC	NC	NC	NC
Intrauterine growth retardation	–	+	–	NC	NC	NC	NC
Short stature	+	+	–	+	–	–	+
Dystrophy	–	+		NC	NC	NC	NC
Upslanted palpebral fissures	–	+	–	NC	NC	NC	NC
Short philtrum	+	+	–	NC	NC	NC	NC
High nasal bridge	–	+	–	NC	NC	NC	NC
Reduced vision	–	+	Unknown	NC	NC	NC	NC
Optic nerve atrophy	–	+	Unknown	NC	NC	NC	NC
Astigmatism	+	+	Unknown	NC	NC	NC	NC
Esophoria	+	+	Unknown	NC	NC	NC	NC
Long, skinny finger	–		–	NC	NC	NC	NC
Syndactyly	–	+	–	NC	NC	NC	NC
Pes adductus	+	+	–	NC	NC	NC	NC
Epilepsy	–	+	N/A	NC	NC	NC	NC
Intellectual disability, moderate	+	+	N/A	–	–	+	+
Delayed speech and language development	+	+	N/A	NC	NC	NC	NC
SRNS	–	–	N/A	+	+	+	+
Muscular hypotonia	+	+	N/A	NC	NC	NC	NC
Cranial MRI abnormalities	+	–	+	–	–	–	–
Abnormality of vision evoked potentials	–	–	N/A	NC	NC	NC	NC

GW, weeks of gestation; MRI, magnetic resonance imaging; SRNS, steroid-resistant nephrotic syndrome; +, yes; –, no; NC, not commented; N/A, not applicable.

a Ravindran et al. (8).

b Braun et al. (4).

To identify the underlying genetic cause of the disease phenotype, we performed the whole genome sequencing (WGS) in the index family and identified compound heterozygous missense mutations in the *NUP85* gene (NM_024844.5) in the index patient: c.454C > A, g.73211897C > A (inherited from mother) and c.487C > A, g.73214291C > A (inherited from father) (Figure 1D). The variant (c.454C > A) has been reported 51 times in heterozygosity in gnomAD (v2.1.1) but has not been reported in homozygosity, while the variant (c.487C > A) has not yet been reported in gnomAD (v2.1.1) and 1,000 Genome. Both variants are predicted to be disease-causative by MutationTaster (www.mutationtaster.org). The CADD phred, SIFT, Polyphen 2, and ClinPred scores for variant c.454C > A were 22.60, 0.093, 0.044, and 0.064 and for variant c.487C > A were 23.40, 0.008, 0.83, and 0.776, respectively. Both the mutations lie in a highly conserved region of the NUP85 protein leading to an exchange of leucine to isoleucine (p.L152I and p.L163I) (NP_079120.1) (Figure 1E). No other variants were identified that met the filtration criteria in the WGS analysis. Western blot analysis on patient-derived fibroblasts revealed the unaltered levels of NUP85 protein between controls and patient samples, indicating the presence of mutant protein (Figures 1F, G) (*n* = 3, one-way ANOVA). Cell viability was significantly reduced in the patient-derived fibroblasts compared to controls (Figure 1H, *n* = 8, one-way ANOVA).

Structural analysis was performed to understand the effect of the identified double mutation (p.L152I and p.L163I) on the structure of the NUP85 protein as well as the NUP107-160 complex and the overall NPC using structural database and prediction tools (15). According to the protein structures from *Homo sapiens*, the two identified mutations are found in close distance to each other, L152I located at the end of helix 3 and L163I at the beginning of helix 4. Structural studies have shown that the Middle Hinge Domain (MHD) is highly conserved and formed by helices four through 13. Since both the mutations L152I and L163I are located at the end and the beginning of two helices with the linker in between as part of the MHD, it is predicted to interfere with the helix arrangement in the MHD region, which in turn alters its orientation and might affect the interaction of NUP85 with NUP214 complex on the cytoplasmic side and NUP205 on the nuclear side of the complex (Figures 1I, J). Overall, these identified mutations are predicted to alter the structure of NUP85 and impair its interactions with the neighboring NUPs and their functions.

## Discussion

In this study, we report compound heterozygous NUP85 variants in an affected individual with MCPH phenotype. In contrast to our previous report on NUP85 variants in two individuals with MCPH-SCKS spectrum disorder, the index patient reported here with *NUP85* variants had only MCPH but no SCKS phenotype (8). Mutations in *NUP37* have been linked to MCPH24 with the clinical phenotype of primary microcephaly, ID, clinodactyly, and cerebellar vermis hypoplasia, but no SRNS (4). Mutations in other NUPs (*NUP107, NUP214*) have also been reported to cause microcephaly in addition to SRNS, whereas mutations in *NUP93, NUP205*, and *NUP160* have been shown to cause SRNS without microcephaly (4). Functional experiments using animal models have revealed that the nature of mutations (hypomorphic or loss-of-function) plays a key role in causing milder phenotypes or severe consequences affecting brain or kidney development (4). In this study, the reported missense variants are located in the highly conserved region of the protein and lead to the unaltered levels of NUP85 protein in the patients indicating the presence of dysfunctional protein. Structural interpretation of the effect of identified variants reveals that the exchange of leucine to isoleucine leads to the reorientation of the MHD, which could interfere with the interacting partners of NUP85 on the nuclear side (NUP205) as well as on the cytoplasmic side (NUP214). These modifications will impact the structural alignment and functioning of the nucleoporins/NPC and thereby affect the cellular processes (16). During brain development, several processes such as proliferation, differentiation, and apoptosis determine the generation of the correct number of neurons and brain size. Any defects in these processes lead to abnormal brain size and function (17). Several NUPs are highly expressed during brain development and play key roles in regulating these cellular processes (1, 18). Several nucleoporins have been shown to exhibit regulatory functions in stem cells during development (19). For example, (i) loss of Nup210 impairs the differentiation of embryonic stem cells to neuroprogenitors (20), (ii) *Nup133* mutant mice are embryonically lethal and they fail to develop terminally differentiated neurons (6), (iii) *Nup50* knockout mice display lethality associated with neural tube defects and intrauterine growth retardation (21), and (iv) knockdown of *Nup153* increases mouse embryonic stem cell differentiation with reduced pluripotency (22). These effects caused by loss/dysfunction of NUPs could be due to the defect in cytoskeletal organization, epigenetic regulation, chromatin architecture, and cell cycle apparatus (19). Components of NPCs (Seh1) are also known to play the key role in the regulation of oligodendrocyte differentiation (23). NUP107-160 complex contributes to proper kinetochore functions during mitosis and is a key for the assembly of bipolar spindles (3, 5). NUP85 localizes to mitotic spindles and its loss causes abnormal mitotic spindles and defective proliferation (3). The underlying pathomechanism of microcephaly has, to a large part, been attributed to defective mitotic machinery affecting the proliferation and/or differentiation of neural precursor cells. Several MCPH-associated genes are known to be key regulators of mitotic spindles and centrosomes (17). NUP85 and several other NUPs have been reported to interact with cytoskeletal structures and nuclear lamins for structural integrity and regulation of gene expression (2). It was shown that mutant NUP85 in patient fibroblasts downregulated the group of cytoskeletal proteins and diminished the actin stress fibers and actin arcs (8). In this study, reduced cell viability of patient fibroblasts might be due to the effect of mutant NUP85 on mitotic spindle morphology and cell cycle process.

In summary, we report an individual with *NUP85* variants with MCPH phenotype, thereby expanding the clinical phenotype spectrum of NUP85-associated diseases and highlighting the role of NUP85 in brain development. Further clinical and functional studies will help to extend the phenotypic spectrum of nucleoporopathies and understand the specific underlying pathomechanism behind the phenotypic variability.

## Data availability statement

The datasets presented in this article are not readily available because of ethical and privacy restrictions. Requests to access the datasets should be directed to the corresponding authors.

## Ethics statement

The studies involving human participants were reviewed and approved by Ethical Committees of the DEFIDIAG project (pilot project of the Plan France Genomique 2025). Written informed consent to participate in this study was provided by the participants' legal guardian/next of kin. Written informed consent was obtained from the minor(s)' legal guardian/next of kin for the publication of any potentially identifiable images or data included in this article.

## Author contributions

AK and ER were responsible for the project conception and drafted the manuscript that was revised and accepted by all co-authors. HM was responsible for the clinical evaluation of the DEFIDIAG study. GL and LJ analyzed and performed the interpretation of WGS data from the DEFIDIAG study. ER and LG performed the experiments and analyzed the data. AD performed the structural simulation and interpretation of data. AK and HM analyzed and interpreted the clinical data.

## Supplementary material

Supplementary Table S1 List of NUP-associated disorders.

Supplementary Figure S1 Height, weight, and occipitofrontal head circumference of index patient.
