# Supplementary material for: Case report: Compound heterozygous NUP85 variants cause autosomal recessive primary microcephaly
Source: Front Neurol. 2023 Feb 9;14:1124886. doi: 10.3389/fneur.2023.1124886 (PMC9947397; doi:10.3389/fneur.2023.1124886)
Supplement: Supplementary Table S1 — List of NUP-associated disorders. [file Data_Sheet_1.docx]

**Supplemental data**

**Table S1. List of NUP-associated disorders**

| **NUP** | **OMIM** | **Phenotype** |
| --- | --- | --- |
| *NUP93* | \|  \| [616892](https://www.omim.org/entry/616892) \| \| --- \| --- \| | Nephrotic syndrome, type 12  Non-progressive congenital ataxia |
| *NUP205* | \|  \| [616893](https://www.omim.org/entry/616893) \| \| --- \| --- \| | Nephrotic syndrome, type 13 |
| *NUP133* | [618349](https://www.omim.org/entry/618349)  [618177](https://www.omim.org/entry/618177) | Galloway-Mowat syndrome 8  Nephrotic syndrome, type 18 |
| *NUP160* | \|  \| [618178](https://www.omim.org/entry/618178) \| \| --- \| --- \| | Nephrotic syndrome, type 19 |
| *NUP85* | \|  \| [618176](https://www.omim.org/entry/618176) \| \| --- \| --- \| | Nephrotic syndrome, type 17  MCPH-SCKS* spectrum disorder |
| *NUP88* | [618393](https://www.omim.org/entry/618393) | Fetal akinesia deformation sequence 4 |
| *NUP155* | [615770](https://www.omim.org/entry/615770) | Atrial fibrillation subtype 15 |
| *NUP107* | [618348](https://www.omim.org/entry/618348)  [616730](https://www.omim.org/entry/616730)   \|  \| [618078](https://www.omim.org/entry/618078) \| \| --- \| --- \| | Galloway-Mowat syndrome 7  Nephrotic syndrome, type 11  Ovarian dysgenesis 6 |
| *NUP214* | [618426](https://www.omim.org/entry/618426)  [601626](https://www.omim.org/entry/601626)  [613065](https://www.omim.org/entry/613065) | Acute infection-induced encephalopathy subtype 9  Leukemia, acute myeloid, somatic  Leukemia, T-cell acute lymphoblastic, somatic |
| *NUP358* | [608033](https://www.omim.org/entry/608033) | Acute Necrotizing Encephalopathy |
| *AAAS* | [231550](https://www.omim.org/entry/231550) | Achalasia-addisonianism-alacrimia syndrome |
| *NUP62* | \|  \| [271930](https://www.omim.org/entry/271930) \| \| --- \| --- \| | Striatonigral degeneration |
| *NUP188* | [618804](https://www.omim.org/entry/618804) | Sandestig-Stefanova syndrome |
| *NUP37* | \|  \| [618179](https://www.omim.org/entry/618179) \| \| --- \| --- \| | Microcephaly 24, primary, autosomal recessive |

*Ravindran E, Juhlen R, Vieira-Vieira CH, Ha T, Salzberg Y, Fichtman B, et al. Expanding the phenotype of NUP85 mutations beyond nephrotic syndrome to primary autosomal recessive microcephaly and Seckel syndrome spectrum disorders. *Hum Mol Genet.* 2021;30(22):2068-81

**Figure S1: Head circumference, body height and weight of index patient.**

**
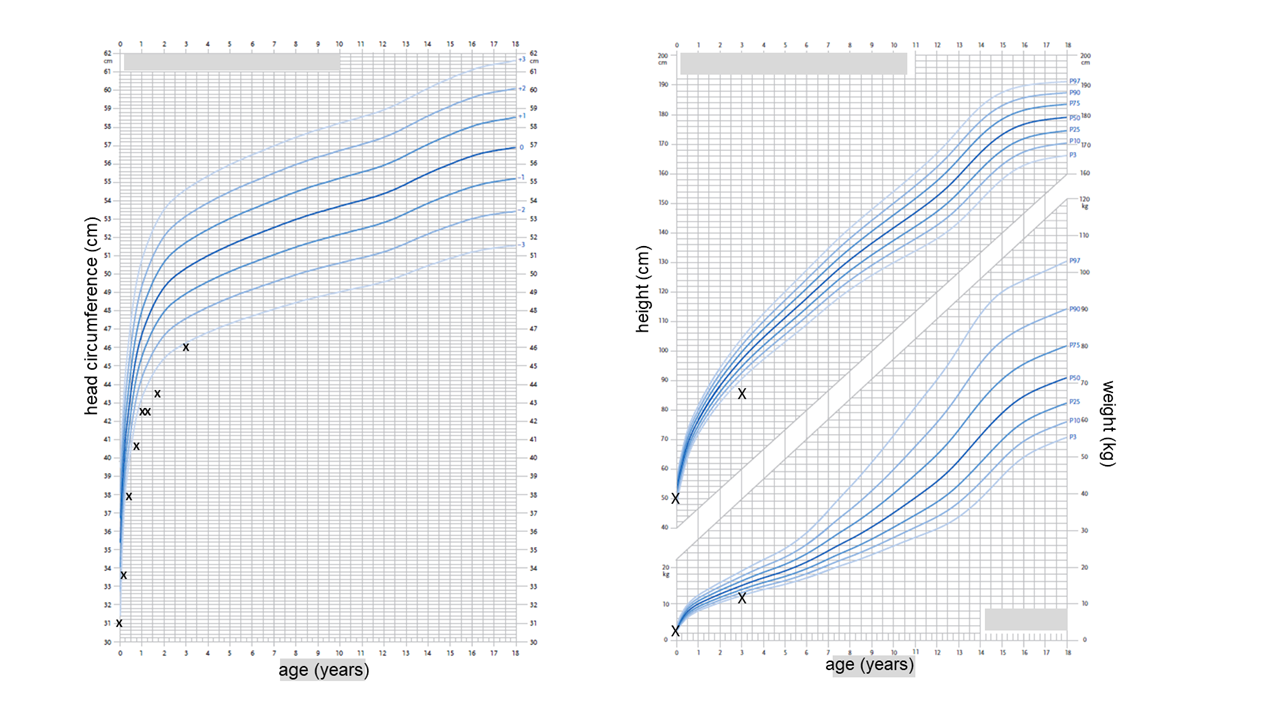
**

**Supplemental methods.**

**Genetic analysis**. The whole genome sequencing trio analysis was carried out within the framework of the DEFIDIAG project (pilot project of the Plan France Genomique 2025) (1). Briefly, patient and parent’s DNA were extracted from blood and sent to the sequencing platform (Centre National de Recherche en Génomique Humaine, CNRGH, Paris). Then 1.1 µg of DNA (Illumina TruSeq PCR free kit) is fragmented using an optimized CNRGH protocol. The whole genome sequencing has been performed by an Illumina HiSeqX5 sequencer using the following parameters: QC follow-up during sequencing (for example: intensity, Q30, phasing / prephasing / bases ratio). The genome sequencing was optimized in order to reach a mean coverage of 30X for each sample. The sequences were aligned to the human reference genome GRCh37 using the Burrows-Wheeler Aligner BWA software and made available as BAM files. Aligned sequences were sorted, cleaned and the PCR duplicates were marked using the Sambamba software (2) in order to eliminate most of the NGS well-known biases. A local realignment of the sequences around insertion and deletion sites and the base quality recalibration was performed using GATK (3). After sequence quality control and alignment of the reference genome, the variant calling on the entire genome for the Single Nucleotide Variants (SNV), small insertion/deletions (indels) and structural variants (including Copy Number Variant, CNV) were performed. SNV and indel calling were performed using the Haplotype Caller from GATK software in “bp resolution” mode to produce gVCF files. Imbalanced SV (CNV) detection > 1 kb was performed using three different softwares: Wisecondor (4), Canvas (5) and Manta (6). Balanced SV (translocation, inversion) detection was done using Manta software. Results were produced in the format of a VCF file to match the common file standard format in NGS analysis. These files were then collected by the IMAGINE Polyweb platform: additional combined TRIO gVCF analysis (genotypeGvcf) and CNV Wisecondor analysis were also performed. The .vcf and .bam files were implemented in Polyweb software developed and previously validated by the IMAGINE bioinformatics platform. The following filtration keys were applied to focus on potentially pathogenic variations: GnomAD allele count < 1,000, GnomAD homozygote count < 5, and predicted protein impact onto all gene transcripts (Stop gain, Stop loss, Start loss, frameshift, in frame deletions or insertions, missense, and predicted splice region, occurrence of this variant in a local non-ID patients cohort is < 1,000 and homozygote count < 5). Ranking of identified variations was then performed based on internal Polyweb criteria: variation sequence quality, de novo status if available, known ID gene or OMIM gene, protein or splicing impact prediction, gene with AR inheritance and homozygous or compound heterozygous variations, male and X linked variation, known pathogenic variations in HGMDpro or ClinVar, frequency in GnomAD. Biological interpretation was performed on the Hospices Civils de Lyon’s genetic laboratory following standard criteria (ACMG recommendations (7)).

**References.**

1. Binquet C, Lejeune C, Faivre L, Bouctot M, Asensio ML, Simon A, et al. Genome Sequencing for Genetics Diagnosis of Patients With Intellectual Disability: The DEFIDIAG Study. Front Genet. 2021;12:766964.

2. Tarasov A, Vilella AJ, Cuppen E, Nijman IJ, Prins P. Sambamba: fast processing of NGS alignment formats. Bioinformatics. 2015;31(12):2032-4.

3. McKenna A, Hanna M, Banks E, Sivachenko A, Cibulskis K, Kernytsky A, et al. The Genome Analysis Toolkit: a MapReduce framework for analyzing next-generation DNA sequencing data. Genome Res. 2010;20(9):1297-303.

4. Raman L, Dheedene A, De Smet M, Van Dorpe J, Menten B. WisecondorX: improved copy number detection for routine shallow whole-genome sequencing. Nucleic Acids Res. 2019;47(4):1605-14.

5. Roller E, Ivakhno S, Lee S, Royce T, Tanner S. Canvas: versatile and scalable detection of copy number variants. Bioinformatics. 2016;32(15):2375-7.

6. Chen X, Schulz-Trieglaff O, Shaw R, Barnes B, Schlesinger F, Kallberg M, et al. Manta: rapid detection of structural variants and indels for germline and cancer sequencing applications. Bioinformatics. 2016;32(8):1220-2.

7. Richards S, Aziz N, Bale S, Bick D, Das S, Gastier-Foster J, et al. Standards and guidelines for the interpretation of sequence variants: a joint consensus recommendation of the American College of Medical Genetics and Genomics and the Association for Molecular Pathology. Genet Med. 2015;17(5):405-24.
